# Selective Brain Network and Cellular Responses Upon Dimethyl Fumarate Immunomodulation in Multiple Sclerosis

**DOI:** 10.3389/fimmu.2019.01779

**Published:** 2019-07-30

**Authors:** Dumitru Ciolac, Felix Luessi, Gabriel Gonzalez-Escamilla, Nabin Koirala, Christian Riedel, Vinzenz Fleischer, Stefan Bittner, Julia Krämer, Sven G. Meuth, Muthuraman Muthuraman, Sergiu Groppa

**Affiliations:** ^1^Department of Neurology, Focus Program Translational Neuroscience (FTN), Rhine-Main Neuroscience Network (rmn^2^), University Medical Center of the Johannes Gutenberg University Mainz, Mainz, Germany; ^2^Department of Neurology, Institute of Emergency Medicine, Chisinau, Moldova; ^3^Laboratory of Neurobiology and Medical Genetics, Nicolae Testemiţanu State University of Medicine and Pharmacy, Chisinau, Moldova; ^4^Department of Neuroradiology, University of Kiel, Kiel, Germany; ^5^Department of Neurology With Institute of Translational Neurology, University of Münster, Münster, Germany

**Keywords:** multiple sclerosis, structural integrity, gray matter networks, white matter networks, immunocellular response, personalized therapy

## Abstract

**Background:** Efficient personalized therapy paradigms are needed to modify the disease course and halt gray (GM) and white matter (WM) damage in patients with multiple sclerosis (MS). Presently, promising disease-modifying drugs show impressive efficiency, however, tailored markers of therapy responses are required. Here, we aimed to detect in a real-world setting patients with a more favorable brain network response and immune cell dynamics upon dimethyl fumarate (DMF) treatment.

**Methods:** In a cohort of 78 MS patients we identified two thoroughly matched groups, based on age, disease duration, disability status and lesion volume, receiving DMF (*n* = 42) and NAT (*n* = 36) and followed them over 16 months. The rate of cortical atrophy and deep GM volumes were quantified. GM and WM network responses were characterized by brain modularization as a marker of regional and global structural alterations. In the DMF group, lymphocyte subsets were analyzed by flow cytometry and related to clinical and MRI parameters.

**Results:** Sixty percent (25 patients) of the DMF and 36% (13 patients) of the NAT group had disease activity during the study period. The rate of cortical atrophy was higher in the DMF group (−2.4%) compared to NAT (−2.1%, *p* < 0.05) group. GM and WM network dynamics presented increased modularization in both groups. When dividing the DMF-treated cohort into patients free of disease activity (*n* = 17, DMF_R_) and patients with disease activity (*n* = 25, DMF_NR_) these groups differed significantly in CD8+ cell depletion counts (DMF_R_: 197.7 ± 97.1/μl; DMF_NR_: 298.4 ± 190.6/μl, *p* = 0.03) and also in cortical atrophy (DMF_R_: −1.7%; DMF_NR_: −3.2%, *p* = 0.01). DMF_R_ presented reduced longitudinal GM and WM modularization and less atrophy as markers of preserved structural global network integrity in comparison to DMF_NR_ and even NAT patients.

**Conclusions:** NAT treatment contributes to a reduced rate of cortical atrophy compared to DMF therapy. However, patients under DMF treatment with a stronger CD8+ T cell depletion present a more favorable response in terms of cortical integrity and GM and WM network responses. Our findings may serve as basis for the development of personalized treatment paradigms.

## Introduction

Loss of structural integrity driven by inflammation, demyelination, and degeneration in multiple sclerosis (MS) involves white matter (WM) and gray matter (GM) compartments, the latter playing a key role in disability and disease progression (1–4). Existing data suggest that CD4+ and CD8+ T cells contribute to the damage of the cortical GM (5, 6). Evaluation of magnetic resonance imaging (MRI)-derived parameters of GM structural alterations was incorporated into studies to track the responses to disease-modifying drugs (DMDs) (7) which have been proven to slow, to various extents, the rate of GM tissue loss. We have recently shown that advanced measures of brain network architecture closely mirror the disease course and clinical impairment (8, 9). Only an exact longitudinal quantification of local and global GM and WM tissue properties enables the development of precise disease course models, thereby creating the basis for personalized therapeutic decisions.

Presently, for relapsing-remitting MS (RRMS) several promising DMDs are available but the long-term benefits of the therapeutic algorithms are still unclear. No unambiguous personalized solutions to halt or ideally reverse the disease course exist; however, first avenues for very efficient immunomodulatory remedies arise. At the same time, markers predicting the favorable response to a specific DMD are under meticulous development but are not yet validated for clinical pathways. Mainly, MRI parameters are regarded as surrogate measures of treatment response to DMDs, although cerebrospinal fluid (CSF) or peripheral blood immune response may also be a valuable biomarker (10). In this respect, treatment response to dimethyl fumarate (DMF) was reflected by reduced counts of CD4+ and CD8+ T cells in patients without disease activity (11–13). Clinically and radiologically stable patients under DMF therapy showed a more pronounced CD8+ than CD4+ T cell reduction compared to active patients (11, 13).

Here, we have postulated that the clinical and brain structural response is tightly linked to the immune cell dynamics under DMF treatment that could be reliably monitored by analyzing peripheral blood lymphocyte subsets. Thus, we aimed to recognize patients with no disease activity and structural deterioration as mirrored by both cortical and subcortical integrity and brain network changes and relate these to immune cell dynamics. A second cohort of patients treated with natalizumab (NAT) served as a reference group to compare clinical, structural and network responses. To this end, we computed regional rates of cortical atrophy, constructed structural GM (from cortical thickness) and WM [from probabilistic tractography (PT)] networks and correlated the atrophy rates with longitudinal changes in lymphocyte subsets.

## Materials and Methods

### Subjects

In this longitudinal study, 78 patients (mean age ± standard deviation (SD) 32.7 ± 8.7 years; 28 males; mean disease duration of 51.1 ± 37.8 months) were selected out of 1,156 patients recruited at the Department of Neurology at the University Medical Center of the Johannes Gutenberg University Mainz in Germany according to the following inclusion criteria: (1) patients aged 18–60 years, (2) patients diagnosed with RRMS, (3) starting DMF or NAT treatment, (4) scanned with a standardized MRI protocol (14), (5) serially acquired MRI scans at several time points, (6) no corticosteroid use within 30 days prior to MRI, (7) peripheral blood samples available at baseline and follow-up time points for DMF-treated patients. Exclusion criteria: (1) necessity in treatment escalation; (2) participation in any interventional trial during the study period; (3) serious adverse events requiring premature study termination; (4) patients with primary or secondary MS progression. Forty-two patients (34.5 ± 9.0 years; 14 males) were identified on DMF treatment (DMF group) and 36 patients (30.6 ± 8.1 years; 14 males) on NAT treatment (NAT group). All patients fulfilled the revised 2010 McDonald diagnostic criteria for RRMS (15). Prior to DMF or NAT therapy patients were exposed to interferon β-1 β, interferon β-1 α or glatiramer acetate. Patients' characteristics are included in Table 1.

**Table 1:** Demographic, clinical, and brain volumetric characteristics of patient groups.

	**DMF group (*****n*** = **42)**		**NAT group (*****n*** = **36)**	
	**Baseline**	**Follow-up**	**Baseline**	**Follow-up**
Age (years)	34.5 ± 9.0		30.6 ± 8.1	
Gender (male/female)	14/28		14/22	
Disease duration (months)	54.2 ± 67.8		47.4 ± 43.9	
Follow-up duration (months)	15.8 ± 7.2		16.1 ± 4.3	
EDSS	1.8 (0–6)	1.7 (0–6)	2.1 (0–6)	2.0 (0–6)
GM volume (mL)	620.0 ± 75.4	617.6 ± 76.1	632.7 ± 73.8	622.4 ± 71.1
WM volume (mL)	572.2 ± 64.7	566.4 ± 46.8	565.9 ± 68.6	561.7 ± 65.5
TB volume (mL)	1434.9 ± 121.4	1427.2 ± 111.9	1448.1 ± 121.2	1433.3 ± 112.3
T2 lesion volume (mL)	11.1 ± 7.5	11.6 ± 8.0	12.8 ± 3.0	12.0 ± 2.5

Variables are presented as means ± SD or median (range).

DMF, dimethyl fumarate; NAT, natalizumab; EDSS, expanded disability status scale; GM, gray matter; WM, white matter; TB, total brain.

*No significant t-tests (p < 0.05) for between or within group comparisons*.

Expanded Disability Status Scale (EDSS) score assessment was obtained at treatment initiation and later at 3-month intervals. MRI data were acquired at the time (1.9 ± 1.7 months) of treatment (DMF/NAT) onset, 6 months after the treatment and then on an annual basis. To track more robust structural and clinical changes, the longest follow-up MRI and EDSS performed after 16 months (15.8 ± 7.2 in the DMF group, 16.1 ± 4.3 in the NAT group) were considered for the analysis. Disease activity was evaluated during the entire course of the study and was based on MRI activity—appearance of new/enlarging T2 lesions or gadolinium-enhancing lesions, and/or on clinical activity—presence of relapse (new neurological symptom not associated with fever/infection, lasting at least 24 h) and sustained disability progression [increase in EDSS by ≥ 1.5 points if the baseline EDSS score was 0, by ≥ 1.0 point if the baseline EDSS score was 1.5, and by ≥ 0.5 points if the baseline EDSS score was > 5.0; (16)].

The study protocol was approved by institutional ethics committee and patients gave written informed consent in accordance with the Declaration of Helsinki.

### Flow Cytometry

In the DMF group, absolute lymphocyte counts and lymphocyte subsets (CD3+, CD4+, CD8+, CD56+, CD19+) were quantified with flow cytometry. Blood samples were collected at baseline (at treatment onset) and later repeatedly with a 6-month interval. Samples collected after almost one and a half years follow-up (15.8 ± 7.2 months) were used for the analysis. Fresh blood samples were drawn into EDTA-containing tubes and exposed to corresponding monoclonal antibodies (BD Biosciences) at room temperature. After erythrocyte lysis and double washing, absolute values of lymphocyte subsets were counted with TruCount beads (BD Biosciences).

### MRI Acquisition

Baseline and follow-up MRI scans were acquired in the study setting with a 32-channel head coil 3T MRI scanner (Magnetom Tim Trio, Siemens Healthcare) according to a standardized protocol (14) comprising sagittal three-dimensional (3D) T1-weighted magnetization prepared rapid gradient echo (MP-RAGE), 3D T2-weighted fluid attenuated inversion recovery (FLAIR) and diffusion tensor imaging (DTI) sequences. Acquisition parameters of applied sequences were: T1 MP-RAGE—repetition time (TR) = 1,900 ms, echo time (TE) = 2.52 ms, inversion time (TI) = 900 ms, echo train length (ETL) = 1, flip angle (FA) = 9°, matrix size = 256 × 256, field of view (FOV) = 256 × 256 mm^2^, slice thickness (ST) = 1 mm; T2-FLAIR – TR = 5000 ms, TE = 388 ms, TI = 1800 ms, ETL = 848, matrix size = 256 × 256, FOV = 256 × 256 mm^2^, ST = 1 mm; DTI – single-shot echo-planar readout, TR = 9000 ms, TE = 102 ms, 30 gradients directions with *b* = 900 s/mm^2^ and one no diffusion image with *b* = 0 s/mm^2^, matrix size = 128 × 128, FOV = 256 × 256 mm, 62 slices, in-plane resolution = 2 × 2 mm^2^, ST = 2.5 mm.

### MRI Processing

**Figure 1:**
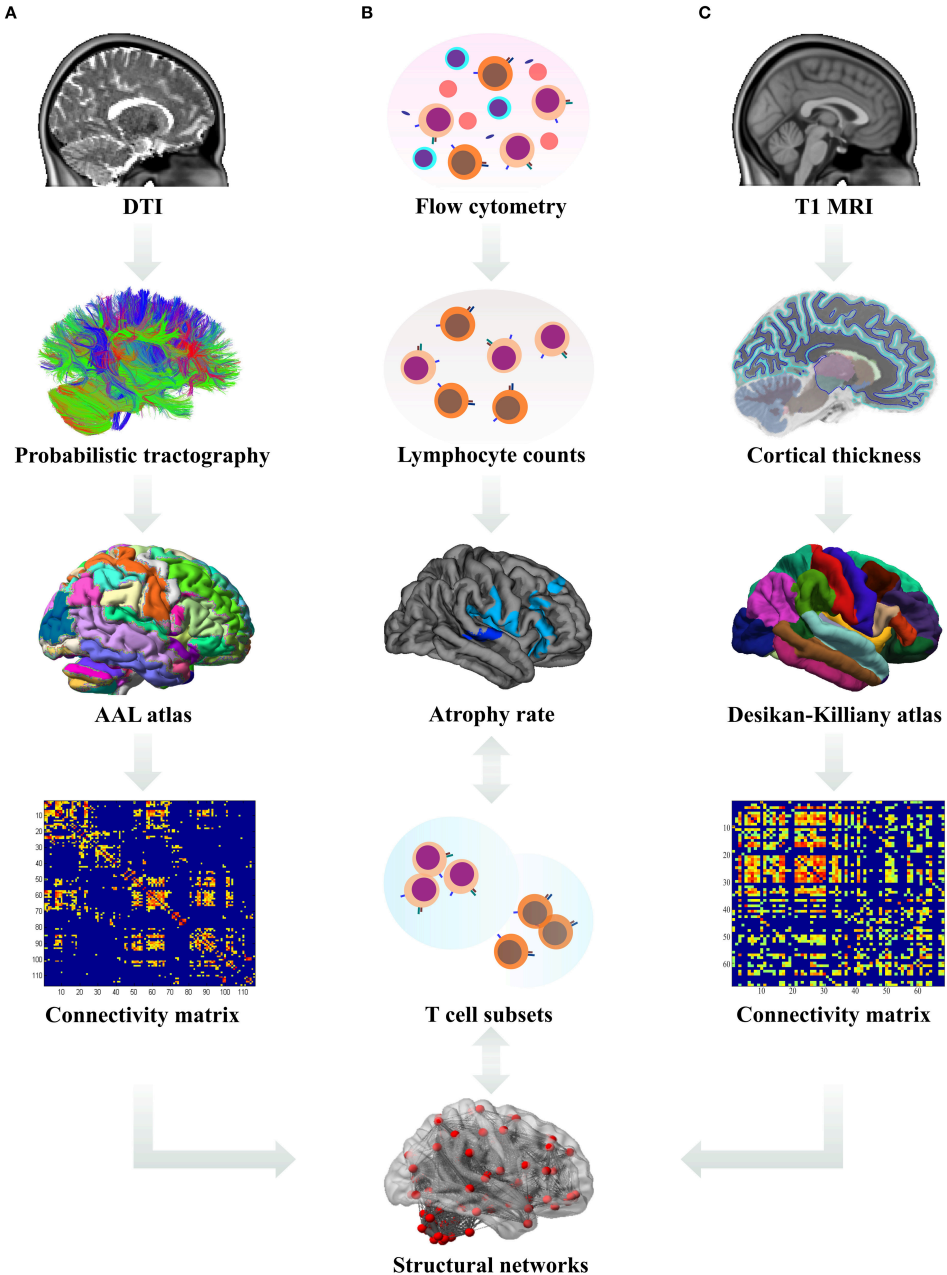
Data analysis pipeline. **(A)** The processed diffusion tensor images (DTI) were used for the derivation of probabilistic tractography. The number of streamlines from each region of interest (ROI, according to the Automated Anatomical Labeling (AAL) atlas) to other ROIs was calculated and a connectivity matrix for each subject was constructed. Subsequently, these white matter derived connectivity matrices were subjected to graph theoretical analysis and the network measures were used in statistical analysis. **(B)** Using flow cytometry, absolute counts of lymphocyte subsets in patient blood samples were estimated and their longitudinal changes correlated with the rates of cortical atrophy; differences in lymphocyte counts between the patient subgroups were analyzed. **(C)** From T1-weighted magnetic resonance images (MRI), cortical thickness for each ROI according to the Desikan–Killiany atlas was calculated and used to estimate the rates of cortical atrophy and construct the connectivity matrices. Subsequently, these gray matter derived connectivity matrices were subjected to graph theoretical analysis and the network measures were used in statistical analysis.

Sequential study pipeline is shown in Figure 1.

### Cortical Thickness: Longitudinal Analysis

Sagittal T1-weighted images were processed using FreeSurfer (version 5.3.0, http://surfer.nmr.mgh.harvard.edu/) (17) for cortical surface reconstruction and volumetric segmentation. The longitudinal pipeline is based on creation of an unbiased within-subject template space and image, using robust inverse consistent registration. Initialization of processing steps—skull stripping, Talairach transformations, atlas registration, and parcellation—runs on the common information from the within-subject template (18–20). Cortical thickness (in mm) was quantified at each vertex of the tessellated surface as the average of the shortest distance between the GM-WM and the GM–CSF interface. All cortical surfaces and subcortical segmentations were manually checked for errors prior to the group analysis. To avoid lesion-induced tissue misclassification errors, gray matter segmentation was performed after filling of T1 hypointense lesions.

Longitudinal changes in cortical thickness between baseline and follow-up were assessed by computing the percent change: thickness at baseline MRI scan was subtracted from the thickness at follow-up MRI scan and divided by the scan interval (in years) and by the average thickness. The resulting surface maps were smoothed with a full width at half maximum (FWHM) Gaussian kernel of 10 mm.

### DTI Analysis

Diffusion tensor computation and tractography analysis was performed using inbuilt functionality in FSL (ver. 5.0.8,http://www.fmrib.ox.ac.uk/fsl); details of this analysis can be found elsewhere (21, 22). In brief, the acquired diffusion data were corrected for subjects' head motion artifacts and eddy current distortions, and subjected to skull and other non-brain tissues removal. Thus, the pre-processed data were then used for computation of the tensor. The distribution of crossing fibers at each voxel of the brain for the computation of PT was estimated using BEDPOSTX (implemented in FSL) and the probability of major and secondary fiber directions was calculated (23). All images were aligned and affine-transformed into the Montreal Neurological Institute (MNI)-152 space. At each voxel a multi-fiber model was fit to the diffusion data, enabling to trace the fibers through regions of crossing or complexity. To obtain an estimate of the probability distribution of connections from each seed voxel 5,000 streamline samples were drawn. The generated tracts are volumes wherein the values at each voxel represent the number of samples (or streamlines) that passed through that particular voxel. Each tract from every seed mask in the atlas was repeatedly sampled (5,000 times) and only those tracts, which passed through at least one other seed mask were retained. For the elimination of spurious connections, tractography in individual subjects was thresholded to include only voxels through which at least 10 percent of all streamline samples had passed.

### Brain Volumes and Lesion Segmentation

Quantification of GM and WM volumes at two time points was done by using voxel-based morphometry (VBM) analysis in Statistical Parametric Mapping (SPM8) software (http://www.fil.ion.ucl.ac.uk/spm). Anatomical 3D T1 and T2-FLAIR images were subjected for spatial normalization, tissue segmentation and spatial smoothing to obtain GM, WM and total brain (TB) volumes (24). Lesion segmentation tool (LST) (version 1.2.3; http://www.applied-statistics.de/lst.html) (25) was used to compute the lesion volume (LV), details of which are mentioned elsewhere (9). Briefly, 3D T2-FLAIR images were co-registered to 3D T1 images and lesion segmentation was run with 20 different thresholds for the lesion growth algorithm (25).

### Graph Theoretical Analysis

#### GM Network Construction

The entire cerebral cortex was parcellated into 68 bilateral anatomical regions of interest (ROIs) (34 ROIs for each hemisphere) based on the Desikan-Killiany atlas (26). Cortical thickness from each cortical ROI was extracted and served for the construction of GM connectivity matrices. These connectivity matrices (size 68 × 68) for each group were obtained by computing the Pearson correlation coefficient between the anatomical regions across the group (8). Graph Analysis Toolbox (GAT) was used to threshold the matrices into multiple densities (ranged from 0.10 to 0.50) and compute the graph theoretical network measures (27).

#### WM Network Construction

The obtained streamlines information from PT (as described above) connecting each pair of ROIs (116—as defined in the Automated Anatomical Labeling (AAL) atlas) was used to construct the connectivity matrix for each subject (28). A more detailed description of the network construction is presented in our previous study (8). The obtained connectivity matrices were included in the graph network analysis for the computation of network properties using Brain Connectivity Toolbox (https://sites.google.com/site/bctnet/) (29).

#### Network Measures

Topological organization of GM and WM networks was assessed by computing the modularity. Modules are groups of nodes forming a distinct subnetwork, where the within module connection (correlation) is higher than the between module correlation (30). Modularity (*Q*) represents the strength of division of the network into modules and was calculated using the Newman's spectral algorithm (31). Since modularity is the measure of networks' segregation, higher modularity indicates more isolated subnetworks within a given network. Hence, with increasing modularity, the long-distance paths between the modules decrease and the local interconnections within the module increase.

#### Statistical Analysis

Statistical analyses were performed using SPSS software (version 23.0; IBM, Armonk, NY, USA.). Normal distribution of the examined data was checked via Shapiro-Wilk test. For a balanced matching of subjects, a multivariate model was tested on our cohort of 1,156 patients with RRMS to select two groups of patients matched upon demographical, clinical and neuroimaging parameters at study entrance.

The H0 hypothesis: we assumed no association between clinical and brain structural responses and immune cell dynamics under DMF treatment. The between-group differences in disease activity (MRI activity, clinical relapse) over the study period were assessed by Pearson's χ^2^-test.

The comparison of lymphocyte counts at baseline and follow-up, and between baseline and follow-up time points in DMF-treated patients, Mann–Whitney *U* and Wilcoxon signed-rank tests were used respectively.

To test if there are any differences in rates of cortical atrophy between patient groups we performed general linear model (GLM) analysis on vertex-by-vertex basis, accounting for the effects of age and gender. Generated statistical parametric maps of significant group differences were corrected for multiple comparisons with Monte Carlo Z permutation cluster analysis (10.000 iterations) at a threshold of Z = 1.3 equivalent to a *p*-value of 0.05. In the DMF group, to assess if there is any relationship between the rate of cortical atrophy and lymphocyte subsets we applied separate GLM models, followed by Monte Carlo Z correction for multiple comparisons as described above.

To examine whether the subcortical volumes vary in time longitudinally as a function of group, a mixed-design repeated-measures ANOVA was performed with *hemisphere* (left and right) and *time* (baseline and follow-up) as within-subject factors and *group* as a between-subject factor with two levels (DMF and NAT), followed by Bonferroni correction.

The GM and WM network measures between baseline and follow-up were compared by paired *t*-test.

## Results

### Subjects

The multivariate analysis revealed no significant differences between the DMF and NAT groups at baseline for age [*F*_(1, 76)_ = 2.75, *p* = 0.10], disease duration [*F*_(1, 76)_ = 0.26, *p* = 0.60], EDSS [*F*_(1, 76)_ = 1.31, *p* = 0.25], lesion volume [*F*_(1, 76)_ = 0.24, *p* = 0.62], GM volume [*F*_(1, 76)_ = 0.56, *p* = 0.45), WM volume [*F*_(1, 76)_ = 0.17, *p* = 0.67], MRI activity [*F*_(1, 76)_ = 2.55, *p* = 0.11] and relapses [*F*_(1, 76)_ = 1.37, *p* = 0.24]. EDSS, GM, WM, TB and T2 lesion volumes at follow-up did not differ significantly from baseline values within the groups (*p* > 0.05), indicating a stable disease period of 16 months on average in these patients despite a 4-year mean disease duration. However, the subgroup analysis within the patients with disease activity showed that the lesion volume was significantly higher at follow-up (12.3 ± 8.2 mL) than at baseline (11.2 ± 8.0 mL, *p* = 0.04) only in the DMF group. During the 16-month follow-up both groups were homogenous in terms of MRI activity (χ^2^ = 2.534, *p* > 0.05) and clinical relapse (χ^2^ = 1.381, *p* > 0.05). In the DMF group, 60% (25 patients) had disease activity [of them 60% (15)—MRI activity, 68% (17)—clinical relapse, 28% (7)—both] and were defined as DMF non-responders (DMF_NR_), while 40% (17 patients) showed no signs of disease activity (DMF responders, DMF_R_). In the NAT group, 36% (13 patients) had disease activity [among them 19% (7) presented MRI activity, 28% (10) had clinical relapse, 11% (4) presented both] and 64% (23 patients) were without disease activity.

During the entire study period, patients with disease activity either from the DMF group or patients from the NAT group didn't have their DMD treatment escalated.

### Peripheral Blood Cell Response Under DMF Treatment

**Figure 2:**
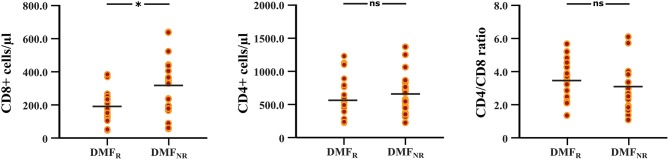
Differences in CD8+, CD4+ T cell counts and CD4/CD8 ratio at follow-up between the DMF responders and DMF non-responders subgroups. DMF responders (DMF_R_) had lower counts of CD8+ T cells at follow-up (after 14.1 ± 5.8 months) in comparison to DMF non-responders (DMF_NR_); *p < 0.05, ns, not significant.

**Figure 3:**
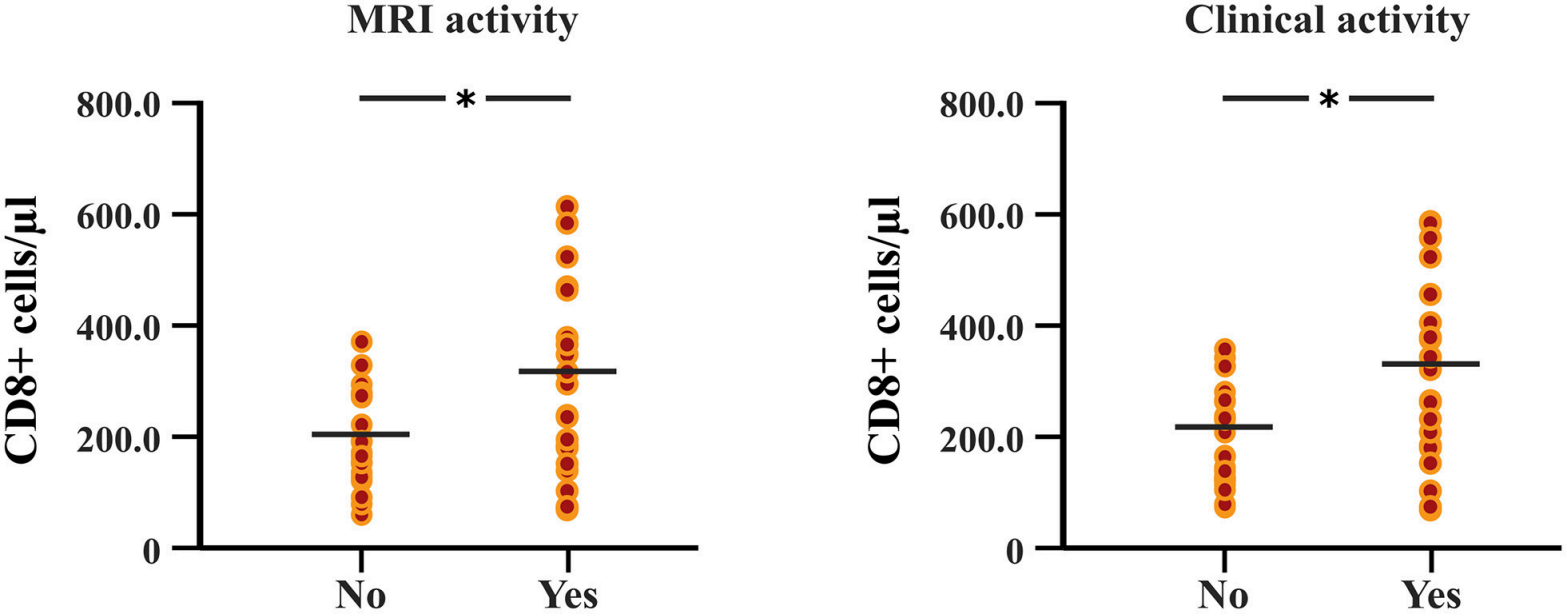
Differences in follow-up CD8+ T cell counts between patients with and without MRI activity, and between patients with and without clinical relapses under DMF treatment. Patients without MRI and clinical activity (DMF responders, DMF_R_) had lower counts of CD8+ T cells at follow-up (after 14.1 ± 5.8 months) in comparison to patients with MRI and clinical activity (DMF non-responders, DMF_NR_); *p < 0.05.

The follow-up period between the baseline and last blood samples for DMF_R_ was 14.1 ± 5.8 months and 16.1 ± 4.9 months for DMF_NR_. Examining the immunological profile of the DMF subgroups, lymphocyte counts did not differ between the DMF_R_ and DMF_NR_ at baseline. At follow-up, DMF-treated patients showed lower counts of CD3+, CD4+, CD8+, CD56+ and ALC, and a higher CD4/CD8 ratio (all *p* < 0.05) compared to baseline, except CD19+, which did not reach significance in DMF_NR_ (*p* > 0.05) (Supplementary Table 1). However, the DMF_R_ subgroup displayed a stronger reduction in CD8+ cells in comparison to the DMF_NR_ (197.7 ± 97.1 /μl vs. 298.4 ± 190.6 /μl; *p* = 0.03) (Figure 2) and a greater change (Δ) in CD8+ cells (−206 /μl vs.-−158 /μl; *p* = 0.01). Similarly, a stronger drop in CD8+ cells was detected separately in patients without MRI activity compared to those with (DMF_R_: 202.3 ± 107.4 /μl vs. DMF_NR_: 312.7 ± 125.0 /μl, *p* = 0.02) and in patients without clinical activity compared to patients with (DMF_R_: 211.1 ± 98.7 /μl vs. DMF_NR_: 327.0 ± 144.5 /μl, *p* = 0.03; Figure 3).

### Cortical Atrophy Rates

**Figure 4:**
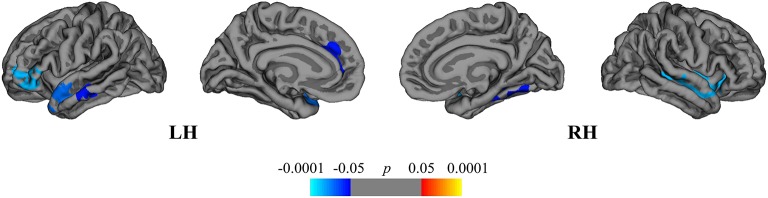
Comparison of cortical atrophy rates between the DMF and NAT groups. Cortical areas displaying the difference in cortical atrophy rates between the DMF and NAT groups, mapped on lateral and medial pial surfaces of the left (LH) and right (RH) hemispheres. Negative values (blue spectrum) denote cortical areas showing greater rates of cortical atrophy in the DMF group in comparison to the NAT group. Color bar indicates the significance levels in the clusters obtained from Monte Carlo simulation at *p* < 0.05 (*Z* = 1.3).

The between-group comparison revealed greater rates of mean cortical atrophy in the DMF group (−2.4%) than in the NAT group (−2.1%, *p* < 0.05), mostly within the frontal and temporal lobes (Figure 4).

**Figure 5:**
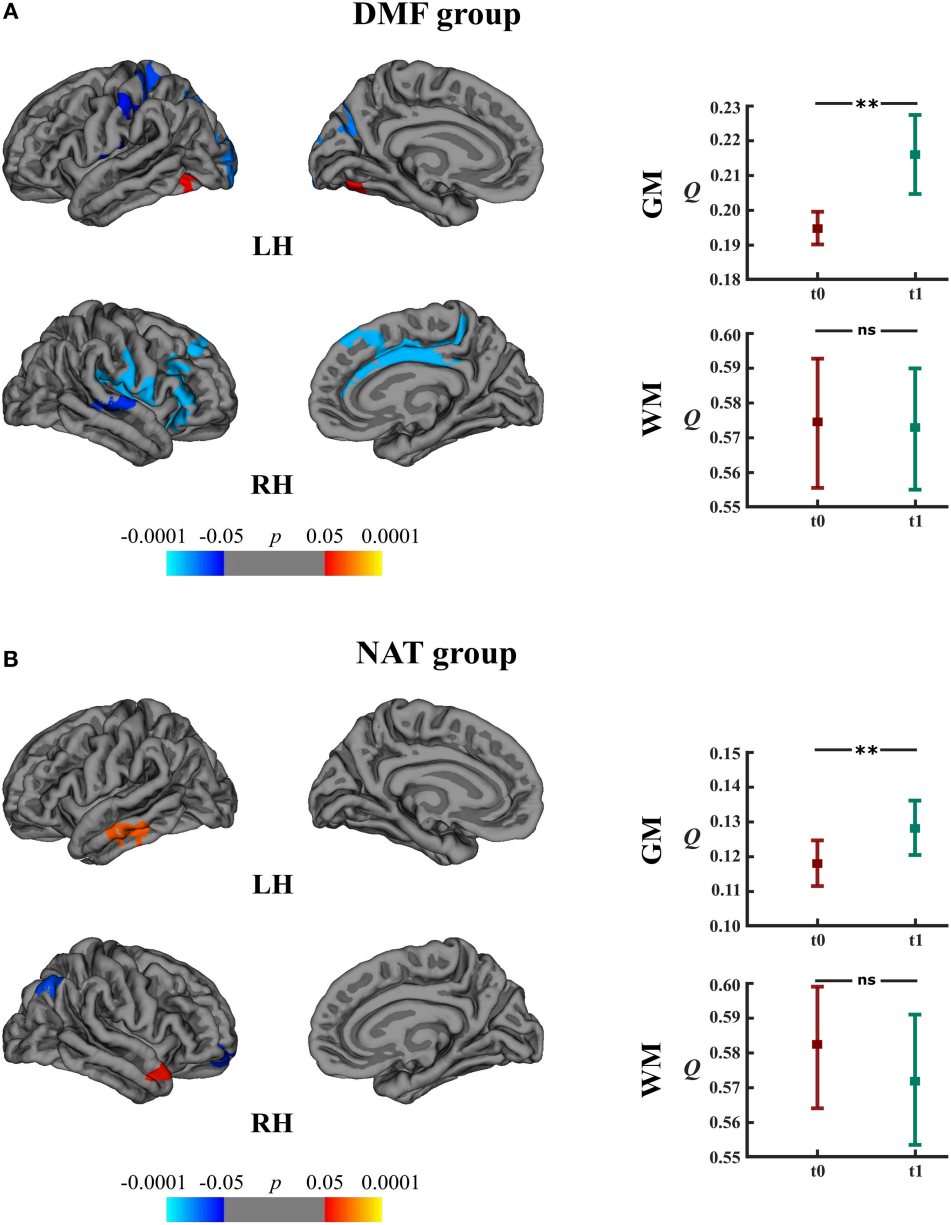
Longitudinal changes in cortical thickness and associated network measures within the DMF and NAT groups. Left: cortical areas displaying the rates of cortical atrophy (negative values, blue spectrum) and cortical thickening (positive values, red spectrum) in the **(A)** DMF and **(B)** NAT groups, mapped on lateral and medial pial surfaces of the left (LH) and right (RH) hemispheres. Color bar indicates the significance levels in the clusters obtained from Monte Carlo simulation at *p* < 0.05 (*Z* = 1.3). Right: modularity (*Q*) of gray matter (GM) and white matter (WM) networks at baseline (t0) and follow-up (t1) in the **(A)** DMF and **(B)** NAT groups; **p < 0.0001, ns, not significant.

Within the DMF group, clusters of cortical atrophy were identified mainly in the frontal, temporal and parietal lobes of both hemispheres (Figure 5A). The NAT group showed clusters of regional cortical atrophy only in the right inferior parietal and rostral middle frontal areas (Figure 5B).

**Figure 6:**
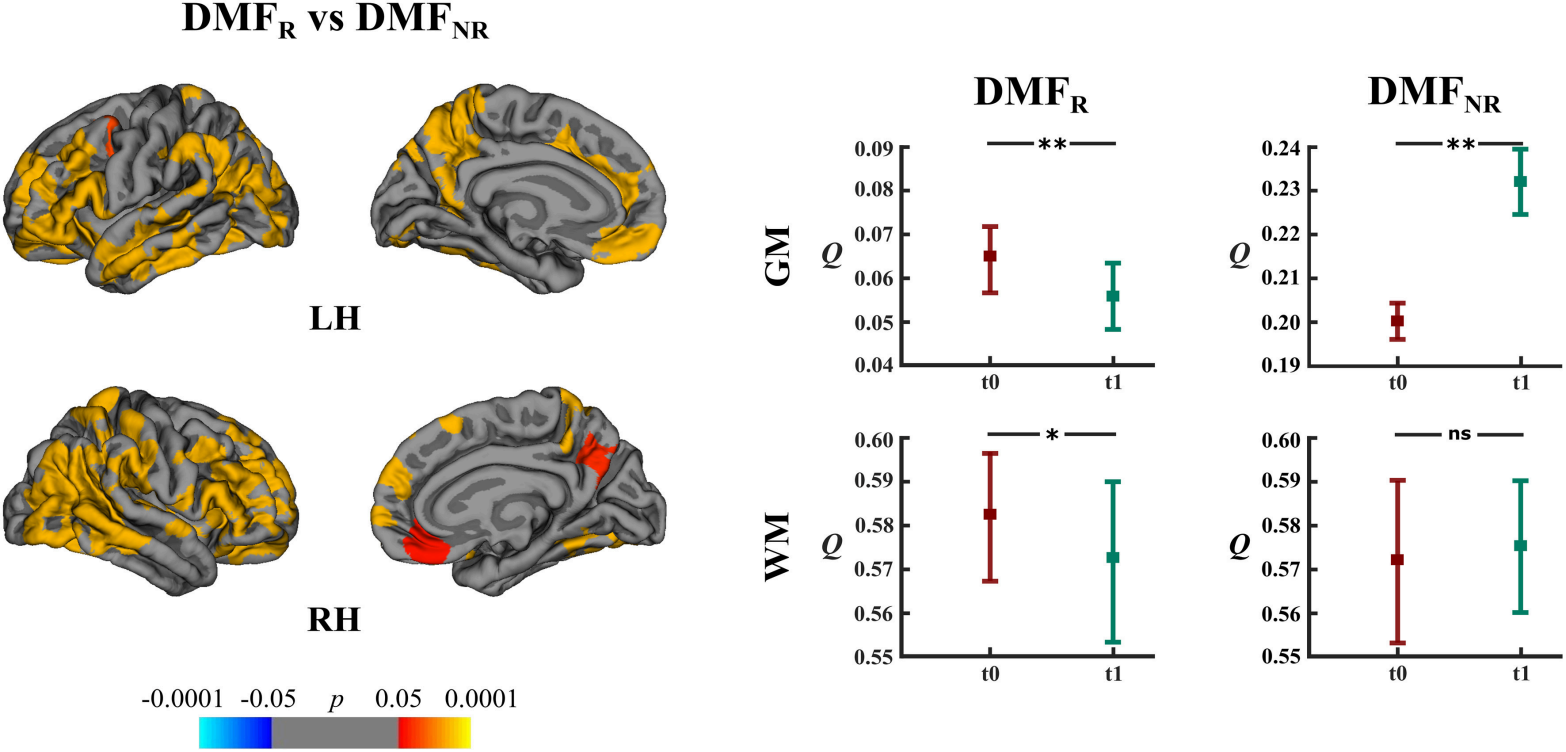
Comparison of cortical atrophy rates between the DMF_R_ and DMF_NR_ subgroups and associated network measures. Left: cortical areas displaying the difference in cortical atrophy rates between the DMF_R_ and DMF_NR_ subgroups, mapped on lateral and medial pial surfaces of the left (LH) and right (RH) hemispheres. Positive values (red spectrum) denote cortical areas showing lower rates of cortical atrophy in the DMF_R_ subgroup in comparison to the DMF_NR_ subgroup. Color bar indicates the significance levels in the clusters obtained from Monte Carlo simulation at *p* < 0.05 (Z = 1.3). Right: modularity (*Q*) of gray matter (GM) and white matter (WM) networks at baseline (t0) and follow-up (t1) in the DMF_R_ and DMF_NR_ subgroups; *p < 0.05, **p < 0.0001, ns, not significant.

DMF_R_ patients had lower mean rates of cortical atrophy in comparison to DMF_NR_ (−1.7% and −3.2%, respectively, *p* < 0.05). The areas showing a subgroup difference in atrophy rates were identified mainly in frontal, temporal and parietal lobes (Figure 6).

**Figure 7:**
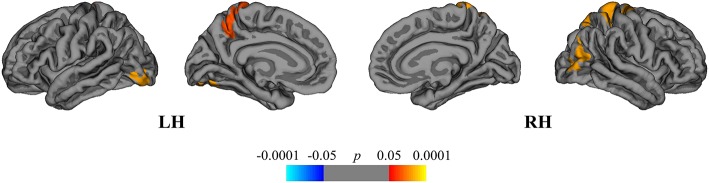
Comparison of cortical atrophy rates between the DMF_R_ and NAT groups. Cortical areas displaying the difference in cortical atrophy rates between the DMF_R_ and NAT groups, mapped on lateral and medial pial surfaces of the left (LH) and right (RH) hemispheres. Positive values (red spectrum) denote cortical areas showing lower rates of cortical atrophy in the DMF_R_ subgroup in comparison to NAT group. Color bar indicates the significance levels in the clusters obtained from Monte Carlo simulation at *p* < 0.05 (*Z* = 1.3).

Finally, we compared the DMF_R_ subgroup with the NAT group in order to evaluate the differences in extent of structural GM loss. Lower regional rates of cortical atrophy were identified in the DMF_R_ subgroup in the following clusters: left lingual, precuneus and right superior, and inferior parietal areas (Figure 7).

Clusters of regional cortical atrophy rates are presented in Supplementary Table 2.

### Relation Between Lymphocyte Subsets and Cortical Atrophy

**Figure 8:**
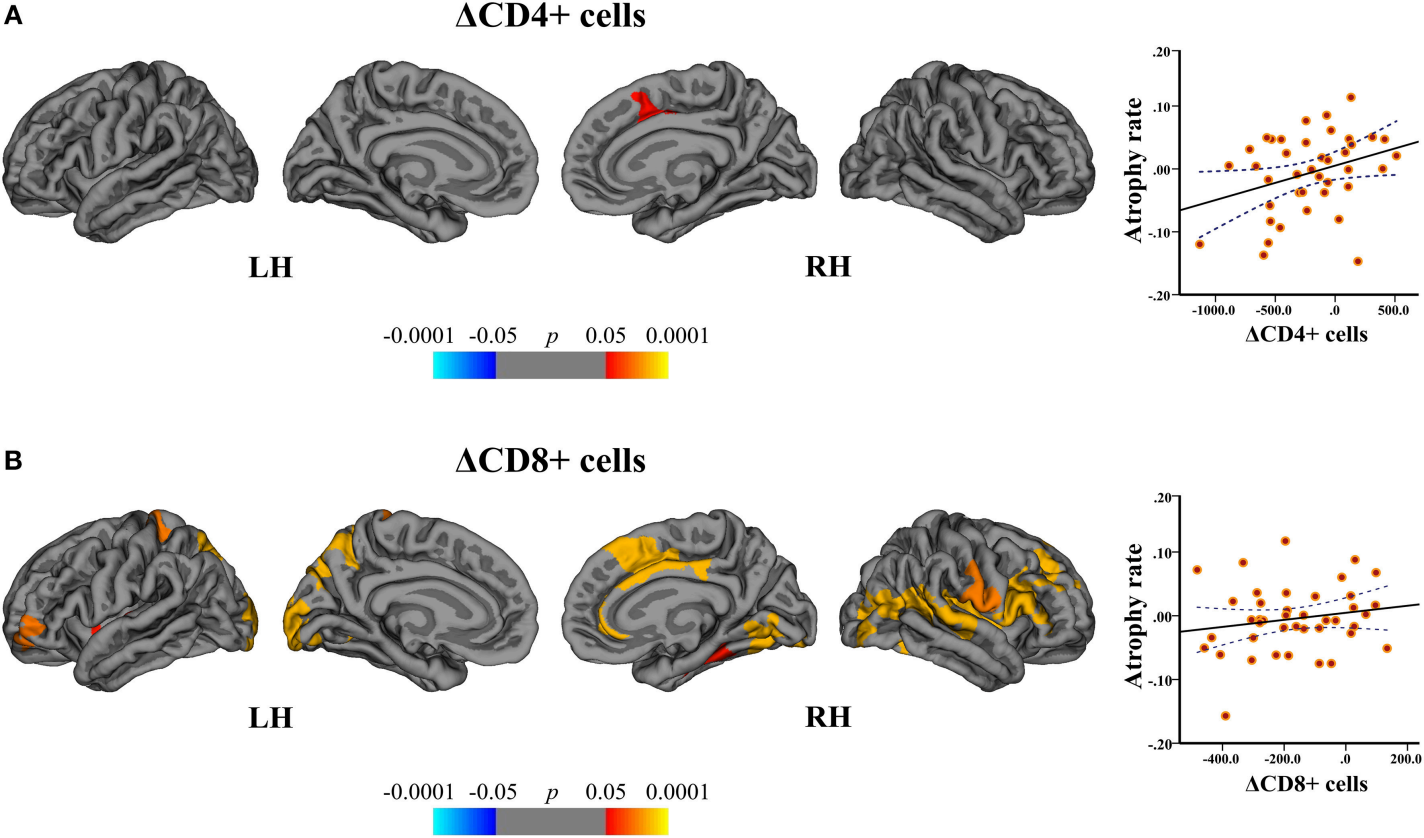
Association between cortical atrophy rates and T cell subsets within the DMF group. Left: cortical areas displaying the correlation between cortical atrophy rates and change (Δ) in **(A)** CD4+ and **(B)** CD8+ cells between baseline and follow-up, mapped on lateral and medial pial surfaces of the left (LH) and right (RH) hemispheres. Color bar indicates the significance levels of the correlation in clusters obtained from Monte Carlo simulation at *p* < 0.05 (*Z* = 1.3). Right: scatter plots showing the association between the cortical atrophy rate in the **(A)** right superior frontal cluster (at peak-vertex) and ΔCD4+ cells and **(B)** left superior parietal cluster (at peak-vertex) and ΔCD8+ cells (dotted line represents the 95% confidence interval for the mean). The decrease in CD4+ and CD8+ T cell counts is associated with the lower rates of cortical atrophy.

In the DMF group, regression analysis disclosed the associations between the cortical atrophy rates, and ΔCD4+ and ΔCD8+ cells between baseline and follow-up. The change in CD4+ cells correlated with the atrophy rate in the right superior frontal area (peak-vertex *R*^2^ = 0.383, *p* = 0.012). The change in CD8+ cells correlated with the atrophy rate in the left superior parietal (peak-vertex *R*^2^ = 0.490, *p* < 0.0001), cuneus (peak-vertex *R*^2^ = 0.583, *p* < 0.0001), and rostral middle frontal (peak-vertex *R*^2^ = 0.489, *p* < 0.0001) and right anterior cingulate (peak-vertex *R*^2^ = 0.518, *p* < 0.0001), lateral occipital (peak-vertex *R*^2^ = 0.531, *p* < 0.0001) and operculum (peak-vertex *R*^2^ = 0.557, *p* < 0.0001) (Figure 8 and Supplementary Table 3). The decrease in CD4+ and CD8+ cells was associated with the lower cortical atrophy rate.

By correlating the rates of cortical atrophy and absolute counts of CD4+ and CD8+ cells similar significant clusters could be obtained.

### Subcortical Structures

Subcortical structures showed no volumetric differences between the DMF and NAT groups (effect of *group, p* > 0.05). There were no differences as well in subcortical volumes accounting for time and hemisphere in both groups (*time* × *hemisphere* × *group* interaction, *p* > 0.05). The same was also true for the DMF subgroups; subcortical volumes did not differ between DMF_R_ and DMF_NR_.

### Gray Matter Network Measures

Longitudinal analysis of GM network measures in the DMF group uncovered increased modularity (*t* = 11.10, *p* < 0.0001) at follow-up (Figure 5A). The number of modules increased from 2 (at baseline) to 5 modules (at follow-up). In the NAT group, modularity (*t* = 5.73, *p* < 0.0001) at follow-up as well was higher than at baseline (Figure 5B) but with less modules at follow-up (2) than at baseline (3).

DMF_R_ subgroup at follow-up displayed lower modularity (*t* = 5.20, *p* < 0.0001) in comparison to baseline (Figure 6). Within the DMF_NR_ subgroup modularity (*t* = 32.11, *p* < 0.0001) at follow-up was higher than at baseline.

### White Matter Network Measures

In both the DMF and NAT groups, WM modularity at follow-up did not differ (*t* = 0.77, *p* = 0.44 and *t* = 1.44, *p* = 0.15, respectively) from the modularity at baseline (Figures 5A,B). Within the DMF group, DMF_R_ at follow-up exhibited lower modularity (*t* = 1.76, *p* = 0.046) than at baseline (Figure 6).

## Discussion

Emerging immunomodulatory therapies considerably modify the individual course of MS, possessing the potential to beneficially influence neuroinflammation and diffuse damage to the GM and WM. However, this therapeutic effectiveness comes at a price of rare, but life-threatening side effects such as the development of secondary immunologic disorders, hematopoietic diseases or progressive multifocal leukoencephalopathy (PML). Thus, individual patients' stratification to therapy response or failure is warranted. Importantly, easy to obtain and measure immunological markers of therapy response are extremely necessary to minimize tissue damage and long-term functional impairment (10, 32). Therefore, here the goal was to stratify patients as responders to DMF therapy by linking cellular responses to clinical, structural MRI and brain network dynamics.

While overall the group of patients treated with NAT displayed less cortical atrophy than patients receiving DMF, the DMF-treated patients free of disease activity with a stronger depletion of CD8+ T cells showed even less GM loss in comparison to the NAT-treated patients. Therefore, we highlight the feasible potential of CD8+ T cell subset monitoring as a marker of individual treatment response. Previous own work (11) and recent emerging data (33–35) showed that lymphocyte subsets present varying susceptibility to DMF. It is worth mentioning that the DMF-induced shifts in lymphocyte subsets cannot be definitely considered as measures of treatment response and currently the reduced counts of CD8+ T and other cells merely explain the DMF mechanism of action. Studies with larger sample size and longer follow-up are required to ascertain if depletion of CD8 (and other lymphocyte subsets) can serve as guiding markers of DMF therapy response and support treatment decisions.

Lymphocyte CD8+ counts correlating with cortical atrophy in patients upon DMF treatment points to the role of CD8+ T cells in ongoing inflammatory processes in GM. As it takes a longer time for DMF in order to achieve a complete effect, a greater loss of cortical GM and disease activity could emerge at first months after DMF treatment onset. Higher cortical atrophy rates in the DMF group could also be caused by inclusion of a higher proportion of patients with disease activity. This is likely to be due to selection bias, since our statistical method to match both patient groups was based not on propensity score methods but on a multivariate model that could possibly over-fit the model and select patients with higher disease activity. Despite a relatively high number of patients with disease activity in the DMF-treated (60%) and in the NAT-treated group (36%), the lesion volume and EDSS didn't significantly change over time. Apparently, this mismatch partly rises from the whole-group analysis because DMF non-responders still showed a significant increase in lesion volume at follow-up. In contrast, available studies evaluating the efficacy of DMF on clinical/MRI activity and brain atrophy measures report a 27% proportion of patients with new relapses, a relative reduction by 21% of disability progression and T2 lesion volume and a reduction by 21% of brain atrophy (36, 37). On the other hand, we are aware that the short follow-up period of our study precludes us to draw definite conclusions on delaying the brain atrophy under both therapies, this being one of the study limitations. As efficacy of DMDs is greater during the second and following years after DMD treatment onset (38–40), our study period of almost one and half years could be relatively enough to obtain approximate impressions on differences of cortical GM and network responses to the DMF and NAT therapy.

In order to quantify discrete structural alterations and depict local and global GM dynamics, we performed longitudinal brain network analysis. This is an emerging tool to explore disease-related reorganization processes that mirror the disease course (8, 9, 41–43). Modularity, a parameter reflecting long-range disconnection and integration of functionally interacting brain regions, is a very sensitive marker of structural integrity in patients with MS (8, 44–46). As the disease progresses in patients with RRMS, the brain circuits reorganize toward a topology of higher modularity with long-range disconnections and local structural homogeneity (9, 44).

With the aid of modularity analysis we found that the GM network dynamics is characterized by increased modularity and long-range disconnections in both DMF- and NAT-treated groups. Longitudinal brain network development toward increased modularity presumably driven by cortical reorganization processes could be an important structural fingerprint mirroring functional impairments or even transition into progressive forms of MS (9). In contrast, we observed an inverse pattern of network topology with decreasing modularity over time in DMF responders, and no differences between the DMF non-responders and NAT patients. Here, we postulate that DMF responders comprise a potentially DMF-induced slowing of neuronal damage and reversal of local and global reorganization processes in the given period.

In both NAT- and DMF-treated groups, WM network topological characteristics did not change over time. On one hand, the stability of WM network topology might be explained by immunomodulatory treatment success. The beneficial impact of NAT on the WM compartment can be assigned to positive effects on myelination, stabilization of the blood-brain barrier and less WM damage (47). On the other hand, the short study period and the methodology applied could have been insufficient to precisely track the WM pathology. The absence of structural alterations in the WM and deep GM compartments possibly suggests different effects of the studied therapies on GM and WM compartments (48). The fact that only DMF responders showed the characteristic decrease in modularity over time highlights a distinct network response of WM networks to DMF exposure as well. We must acknowledge that the conclusions drawn from the WM network analysis need to be interpreted with caution, since mapping of white matter connections by diffusion MRI tractography has inherent sources of errors, artifacts and biases that limit its anatomical accuracy and the validity of obtained estimates (49). WM lesions may lead to inaccurate tracking of termination sites of fibers or even cause deviations of fiber bundles nearby the lesions (50). The DTI protocol and computational algorithms used in this study can partly overcome the constraints posed by the microstructural complexity of WM tracts (fibers with crossing configurations, geometric distortion, folding patterns) (51).

## Conclusions

Our results indicate that NAT therapy opposed to DMF treatment favors preservation of cortical and subcortical structural integrity but with equivalent network responses. But within the patient cohort treated with DMF, a more pronounced decline in circulating CD8+ T lymphocytes was associated with a favorable clinical outcome and advantageous structural network responses. Whether DMF could serve as a treatment strategy in NAT-incompatible patients under conditions of rigorous T cell monitoring should be further investigated.

## Ethics Statement

The study protocol was approved by institutional ethics committee and patients gave written informed consent in accordance with the Declaration of Helsinki.

## Author Contributions

Conceived and designed the study: SM, MM, and SG. Acquired and analyzed patients' data: DC, FL, GG-E, NK, CR, VF, JK, and MM. Interpreted the data and drafted the manuscript: DC, FL, SB, SM, MM, and SG. Interpreted the data and revised the manuscript: VF, SM, MM, and SG.

### Conflict of Interest Statement

The authors declare that the research was conducted in the absence of any commercial or financial relationships that could be construed as a potential conflict of interest.
