# Supplementary material for: Selective Brain Network and Cellular Responses Upon Dimethyl Fumarate Immunomodulation in Multiple Sclerosis
Source: Front Immunol. 2019 Jul 30;10:1779. doi: 10.3389/fimmu.2019.01779 (PMC6682686; doi:10.3389/fimmu.2019.01779)
Supplement: Supplementary file 1 [file Table_1.DOCX]

**Supplementary Table 1.** Absolute counts of lymphocyte subsets in the DMF subgroups at baseline and follow-up.

|  | **DMF_R_ subgroup (n=17)** | | **DMF_NR_ subgroup (n=25)** | |
| --- | --- | --- | --- | --- |
|  | **baseline** | **follow-up** | **baseline** | **follow-up** |
| **CD3**+ **(/µl)** | 1391.9±477.7 | 990.7±472.0^a^ | 1406.1±624.6 | 887.7±432.7^a^ |
| **CD4**+ **(/µl)** | 871.8±320.5 | 655.6±317.1^a^ | 935.5±474.6 | 669.4±356.7^a^ |
| **CD8**+ **(/µl)** | 403.6±199.2 | 197.7±97.1^a^ | 457.3±224.0 | 298.4±190.6^a^ |
| **CD56**+ **(/µl)** | 178.3±117.3 | 103.7±49.0^a^ | 226.3±128.6 | 156.0±75.7^a^ |
| **CD19**+ **(/µl)** | 237.7±137.6 | 170.2±84.2^a^ | 291.4±239.8 | 170.2±82.0 |
| **CD4/CD8 ratio** | 2.4±1.0 | 3.5±1.2^a^ | 2.2±1.2 | 2.8±1.5^a^ |
| **ALC (/µl)** | 1822.4±676.5 | 1178.2±439.0^a^ | 2078.6±763.2 | 1374.6±471.5^a^ |
| Cell counts are presented as mean±SD.  ^a^Significant at *p*<0.05 (Wilcoxon signed-rank test between baseline and follow-up values).  Abbreviations: DMF_R_ = dimethyl fumarate responders; DMF_NR_ = dimethyl fumarate non-responders; ALC = absolute lymphocyte count. | | | | |
