# Supplementary material for: Selective Brain Network and Cellular Responses Upon Dimethyl Fumarate Immunomodulation in Multiple Sclerosis
Source: Front Immunol. 2019 Jul 30;10:1779. doi: 10.3389/fimmu.2019.01779 (PMC6682686; doi:10.3389/fimmu.2019.01779)
Supplement: Supplementary file 2 [file Table_2.DOCX]

**Supplementary Table 2.** Clusters showing significant regional rates of cortical atrophy. Corresponding cluster-wise *p*-values (CWP), cluster size (mm^2^) and Talairach (Tal X, Y, Z) coordinates.

| **Cluster**^a^ **No** | **CWP** | **Size (mm^2^)** | **Tal X** | **Tal Y** | **Tal Z** | **Cortical area** |
| --- | --- | --- | --- | --- | --- | --- |
| **DMF group** | | | | | | |
| Left hemisphere | | | | | | |
| **1**  **2**  **3**  **4**  **5** | 0.0007  0.0007  0.0047  0.0240  0.0472 | 933.74  944.19  731.20  583.34  527.19 | -11.4  -20.2  -20.4  -54.4  -33.5 | -72.0  -93.5  -35.8  -6.2  -66.5 | 21.1  8.3  56.9  9.0  -9.5 | cuneus  lateral occipital  postcentral  precentral  fusiform |
| Right hemisphere | | | | | | |
| **1**  **2**  **3**  **4** | 0.0001  0.0001  0.0001  0.0091 | 2817.95  1712.02  2285.93  699.53 | 6.3  45.8  42.6  61.4 | 25.8  30.8  -32.4  -12.5 | 18.8  -3.8  24.1  2.2 | anterior cingulate  pars triangularis  supramarginal  superior temporal |
| **NAT group** | | | | | | |
| Left hemisphere | | | | | | |
| **1** | 0.0024 | 802.40 | -52.2 | -34.0 | -13.6 | inferior temporal |
| Right hemisphere | | | | | | |
| **1**  **2**  **3** | 0.0028  0.0119  0.0235 | 774.24  657.86  593.49 | 39.2  30.3  45.8 | -59.2  52.0  8.7 | 45.4  -8.4  -19.6 | inferior parietal  rostral middle frontal  superior temporal |
| **DMF group vs NAT group** | | | | | | |
| Left hemisphere | | | | | | |
| **1**  **2**  **3**  **4** | 0.0000  0.0007  0.0214  0.0247 | 1177.15  879.06  585.87  575.48 | -46.8  -36.8  -12.8  -52.7 | 32.4  6.1  32.6  -24.2 | -6.6  -26.3  17.2  -10.3 | pars triangularis  temporal pole  pars triangularis  middle temporal |
| Right hemisphere | | | | | | |
| **1**  **2** | 0.0001  0.0276 | 2009.11  581.47 | 35.0  28.2 | -7.4  -59.0 | -4.2  -8.5 | insula  fusiform |
| **DMF_R_ subgroup vs DMF_NR_ subgroup** | | | | | | |
| Left hemisphere | | | | | | |
| **1**  **2**  **3**  **4**  **5**  **6** | 0.0001  0.0001  0.0001  0.0001  0.0001  0.0042 | 3342.16  7524.04  5305.81  7605.12  2131.45  748.66 | -14.2  -29.4  -55.0  -30.0  -10.1  -30.5 | -47.5  45.6  -11.0  -61.3  31.0  9.9 | 36.0  5.5  -18.2  40.4  26.5  50.1 | precuneus  rostral middle frontal  middle temporal  inferior parietal  superior frontal  caudal middle frontal |
| Right hemisphere | | | | | | |
| **1**  **2**  **3**  **4** | 0.0001  0.0001  0.0111  0.0223 | 25497.87  1273.47  679.28  608.52 | 40.7  26.0  10.5  5.5 | -61.2  54.7  -52.6  36.8 | 10.4  -12.4  38.2  -18.6 | inferior parietal  rostral middle frontal  precuneus  medial orbitofrontal |
| **DMF_R_ subgroup vs NAT group** | | | | | | |
| Left hemisphere | | | | | | |
| **1**  **2** | 0.0001  0.0024 | 1154.51  799.41 | -19.5  -11.2 | -83.2  -52.3 | -3.5  45.3 | lingual  precuneus |
| Right hemisphere | | | | | | |
| **1**  **2** | 0.0001  0.0001 | 1939.97  979.14 | 25.9  40.5 | -41.5  -64.7 | 60.4  14.6 | superior parietal  inferior parietal |
| ^a^Clusters corrected for multiple comparison using family-wise error correction with Monte Carlo Z simulation at *p*<0.05 (Z=1.3).  Abbreviations: DMF = dimethyl fumarate; NAT = natalizumab; DMF_R_ = DMF responders; DMF_NR_ **=** DMF non-responders. | | | | | | |
