# Supplementary material for: Selective Brain Network and Cellular Responses Upon Dimethyl Fumarate Immunomodulation in Multiple Sclerosis
Source: Front Immunol. 2019 Jul 30;10:1779. doi: 10.3389/fimmu.2019.01779 (PMC6682686; doi:10.3389/fimmu.2019.01779)
Supplement: Supplementary file 3 [file Table_3.DOCX]

**Supplementary Table 3.** Clusters showing the correlations between the rate of cortical atrophy and change (Δ) in T cell subsets between baseline and follow-up. Corresponding cluster-wise p-values (CWP), cluster size (mm2), and Talairach (Tal X, Y, Z) coordinates.

| **Cluster**^a^ **No** | **CWP** | **Size (mm^2^)** | **Tal X** | **Tal Y** | **Tal Z** | **Cortical area** |
| --- | --- | --- | --- | --- | --- | --- |
| **ΔCD4+** | | | | | | |
| Left hemisphere | | | | | | |
| no clusters | | | | | | |
| Right hemisphere | | | | | | |
| **1** | 0.0336 | 562.14 | 12.1 | 11.8 | 39.2 | superior frontal |
| **ΔCD8+** | | | | | | |
| Left hemisphere | | | | | | |
| **1**  **2**  **3**  **4**  **5** | 0.0001  0.0001  0.0001  0.0218  0.0333 | 2147.21  2351.79  885.05  593.68  559.22 | -10.1  -4.9  -33.3  -49.6  -35.47 | -81.4  -91.3  50.3  -14.3  -79.3 | 35.6  14.7  -0.5  2.2  -8.3 | superior parietal  cuneus  rostral middle frontal  transverse temporal  lateral occipital |
| Right hemisphere | | | | | | |
| **1**  **2**  **3**  **4**  **5** | 0.0001  0.0001  0.0001  0.0001  0.0223 | 2342.98  8458.47  2309.52  1053.59  610.02 | 8.8  25.0  36.5  26.7  37.4 | 8.7  -86.0  16.9  35.1  -36.1 | 34.2  -3.2  19.3  22.8  -10.5 | anterior cingulate  lateral occipital  pars opercularis  rostral middle frontal  fusiform |
| ^a^Clusters corrected for multiple comparison using family-wise error correction with Monte Carlo Z simulation at a *p*<0.05 (Z=1.3).  Abbreviations: ΔCD4+ = change in CD4+ cells; ΔCD8+ = change in CD8+ cells. | | | | | | |
